# Nurse managers' perceptions and experiences of caring behavior for clinical nurses: a multicenter survey

**DOI:** 10.1186/s12912-023-01541-0

**Published:** 2023-10-16

**Authors:** Lulu Liao, Fengjian Zhang, Yan Zhang, Chunyan Guan, Guihua Xu, Changrong Yuan, Xiufen Yang, Lei Huang, Wei Wang, Xiaoxiao He, Juan Xu, Yilan Liu

**Affiliations:** 1 Department of Nursing, Union Hospital, Tongji Medical College, Huazhong University of Science and Technology, Wuhan, Hubei China; 2 School of Nursing, Tongji Medical College, Huazhong University of Science and Technology, Wuhan, Hubei China; 3 School of Medicine and Health Management, Huazhong University of Science and Technology, Wuhan, Hubei China; 4 School of Nursing, Nanjing University of Chinese Medicine, Nanjing, Jiangsu China; 5 School of Nursing, Fudan University, Shanghai, China; 6 Department of Geriatric, Shenzhen People’s Hospital (The First Affiliated Hospital, Southern University of Science and Technology), Shenzhen, Guangdong, China

**Keywords:** Humanistic care, Nursing quality, Nurse managers, Mixed methods

## Abstract

**Background:**

Humanistic care management is a necessary measure to improve the motivation and initiative of clinical nurses and is the foundation to improve the quality of nursing. Understanding the current status and identifying the influencing factors that promote or hinder humanistic care behaviors is essential. This study investigated the current status and experiences of nurse managers' caring behaviors toward clinical nurses.

**Methods:**

We conducted a mixed-methods study with an explanatory sequential design. A survey on the nurse managers' caring behaviors in 101 hospitals from 23 provinces and four municipalities in China was investigated (*n* = 2022). Then, semi-structured interviews were conducted to obtain information about the participants’ experiences associated with the performance of caring behaviors (*n* = 27).

**Results:**

Survey data demonstrated that the nurse managers' overall caring behaviors were moderately good. The total scoring rate was 88.55%, and the overall score was 161.19 ± 20.68. Qualitative data revealed that the capacity of nurse managers and clinical nurses, opportunity, and motivation to implement humanistic care are key influencing factors of caring behaviors.

**Conclusions:**

The results suggested that intrinsic motivation, organizational support, and the humanistic care capabilities of clinical nurses and nurse managers are vital to implementing care behaviors. Thus, successful humanistic care management requires a concerted effort at the individual and organizational levels.

**Supplementary Information:**

The online version contains supplementary material available at 10.1186/s12912-023-01541-0.

## Background

Humanistic care is essential to providing quality nursing services [1]. Valuing human beings is foundational to the nursing profession and the essence of care. As early as 1988, Watson proposed that humanistic care is an active willingness or responsibility to care for people, which could be effectively expressed through interpersonal interactions [2]. Humanistic management theory emphasizes integrating humanistic care into management, encouraging individuals to pursue fulfillment and realize self-worth in their work [3], which is essential to the smooth running of daily work.

Clinical nurses comprise the largest component of the specialized healthcare workforce in hospitals, providing first-line care [4]. Clinical nurses often suffer from work stress and psychosocial problems due to the hospital's fast-paced work environment [5]. Nurses faced physical stress and tremendous moral pressure, especially during the coronavirus 2019 (COVID-19) pandemic [6]. It has been suggested that clinical nurses are often exposed to workplace bullying, leading to job dissatisfaction and leaving the profession [7]. Studies have shown that job burnout is a common problem among nurses, seriously affecting their emotional state, health status, and medical quality. However, the public ignores nurses' pressure, and everyone focuses on the patients [8]. It is believed that clinical nurses with more powerful leadership support can more effectively relieve stress, sort out conflict, and increase their ability to work under pressure [9]. In Chinese hospitals, a large part of the department's resources is controlled by nurse managers, who are the most direct supervisors. The benefits of effective leadership of nurse managers are attributed to increased psychological empowerment of nurses, resulting in positive outcomes [10].

As clinical front-line managers, nurse leaders serve as role models, which may affect the professional values of clinical nurses [11]. Moreover, through the humanistic care delivery chain, front-line nursing staff feel the care of nurse managers and pass it on to patients and their colleagues, which is more conducive to nursing quality [12]. Humanistic care for clinical nurses has become the most crucial and indispensable aspect of management.

There are several gaps in the existing literature. Some studies have examined the impact of nurse managers' caring behaviors on the working environment and negative behaviors (e.g., workplace bullying), which have been quantitative surveys with small sample sizes [13, 14]. A few studies have used a qualitative design to explore clinical nurses' perception of caring behavior towards nursing managers [15]. Most studies have been conducted from the perspective of clinical nurses and rarely from the perspective of nurse managers. These interviewees are from the same hospital and do not represent the population. Based on a significant population in China, no large population research has been conducted on nurse managers' perceptions and experiences of caring behaviors for clinical nurses.

Swanson's caring theory includes five elements: Knowing, Being with, Doing for, Enabling, and Maintaining belief, which has been used in a large number of clinical practices in the field of nursing [16]. The present study developed the interview outline based on Swanson’s theory of caring to explore the caring behaviors of nurse managers. Caring behaviors are a complex social process that develops from commitments in interpersonal relationships [17]. The capability, opportunity, motivation, and behavior (COM-B) model is a holistic framework that in order to instigate behavior change, individuals must be capable of undertaking it, be presented with opportunities, and be motivated to engage with the behavior actively [18]. We used the COM-B model to guide data analysis and elaborate on the barriers and enablers nurse managers encounter in changing their caring behavior.

By mixing quantitative and qualitative studies, researchers gained breadth and depth of understanding [19]. Altogether, different methods can achieve complementary advantages and multiple crossovers, and the results can be mutually verified and interpreted. Based on a multicenter national survey, this study aimed to elucidate the level of nurse managers' caring behaviors. Furthermore, this study sought to explore, from the nurse managers' perspective, what barriers and enablers to humanistic care exist.

## Methods

### Design

**Fig. 1:**
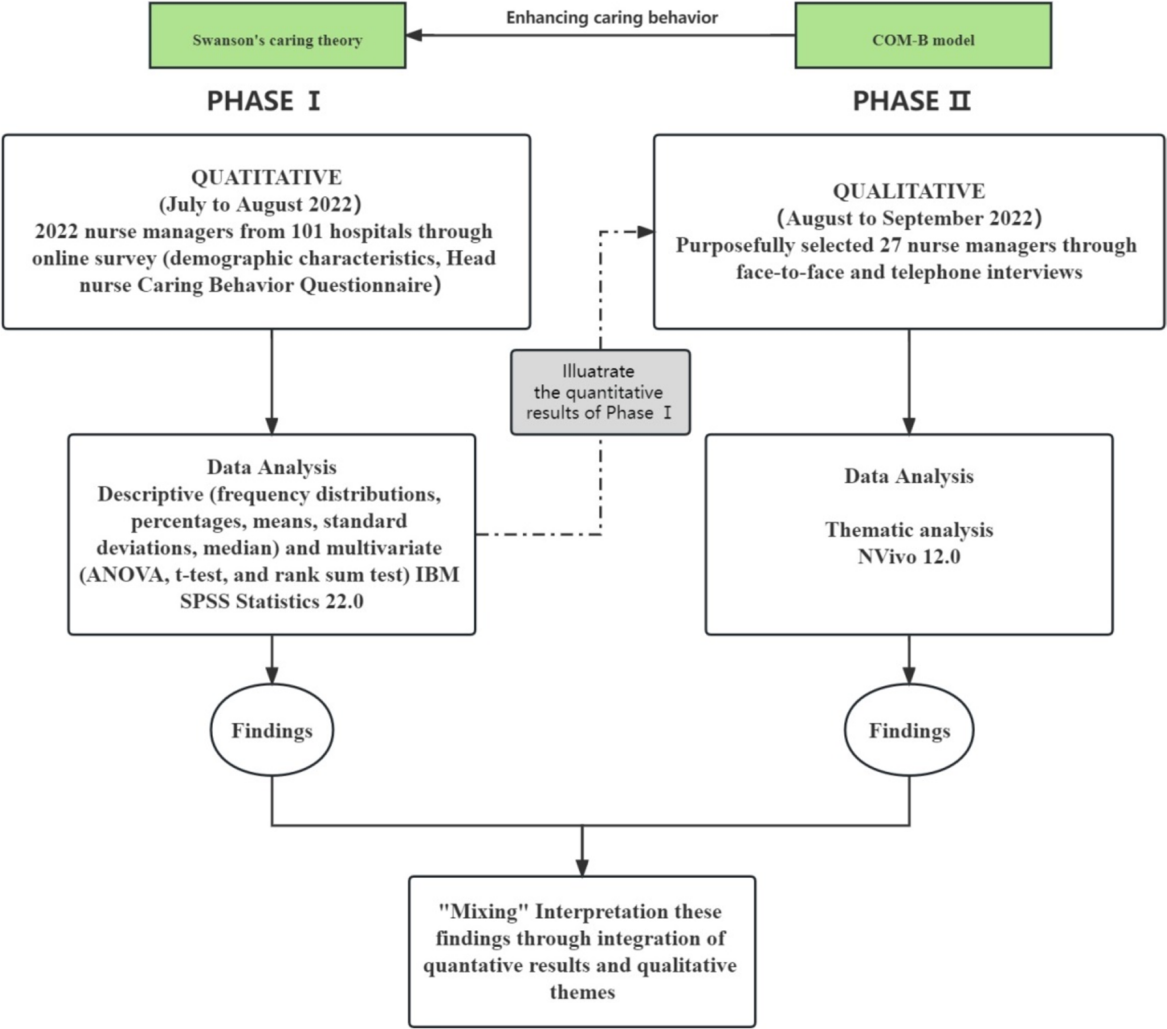
Flowchart of explanatory sequential study design

This study adopted a sequential explanatory mixed method approach comprising two evaluation phases (Fig. 1) [20]. This method used qualitative findings to help explain and better describe information related to the quantitative data. It was selected as it allowed us to explore the challenges and enablers nurse managers faced when implementing humanistic care. The good reporting of a mixed methods study (GRAMMS) checklist [21] was observed in the reporting of this article (File S1).

**Fig. 2:**
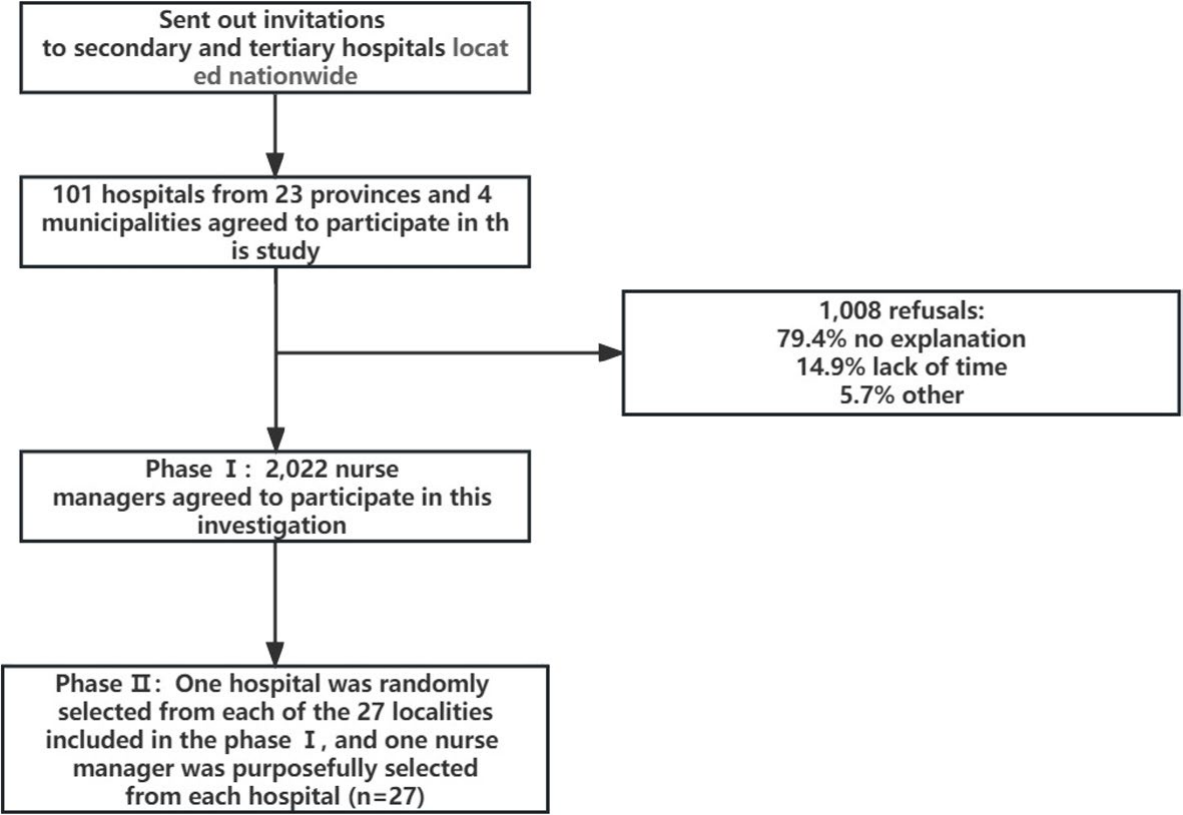
Flowchart of sampling procedures

The researchers recruited hospitals through the Humanistic Nursing Professional Committee of the Chinese Vital Care Association. The Humanistic Nursing Professional Committee of China Life Care Association led the development of the expert consensus on the Norms of Practice of Humanistic Care in Hospital Nursing and the group standard (Registration number: IPGRP-2022CN073) [22], the Management Norms of Humanistic Care in Ward Nursing (T/CALC 001–2022). This study is part of a preliminary baseline survey for promoting and applying this consensus and group standard in secondary and tertiary hospitals in China, focusing on the current situation of nurse managers' caring behaviors towards clinical nurses. Figure 2 depicts the sampling flowchart.

### Participants

First, the Humanistic Nursing Professional Committee of China Life Care Association issued the recruitment notice. All enrolled hospitals that met the following inclusion and exclusion criteria were eligible for inclusion in this study. Table 1 presents the inclusion and exclusion criteria used for this study. In the phase of the quantitative study, we included all nurse managers who fulfilled the inclusion criteria and did not meet the exclusion criteria. The nursing manager interviewed for the qualitative phase was recommended by the director of the nursing department in this hospital, who has extensive experience with humanistic care management.

**Table 1:** Inclusion and exclusion criteria of the hospitals and nurse managers

	Inclusion Criteria	Exclusion Criteria
Hospitals	(i) Secondary or tertiary hospitals; (ii) It is willing to be a pilot unit to promote the expert consensus on the practice norms of humanistic care in the hospital; (iii) Voluntarily joined in this study	(i) Other related programs are being implemented in the hospital
Nurse managers	(i) Participants voluntarily participated in the survey; (ii) Worked as a ward manager; (iii) Holding nurse practice certificate; (iv) Years of working as a nurse manager ≥ 1 year	(i) Unable to understand the purpose of this study

### Instruments

The survey questionnaire consisted of demographic characteristics of the Nurse Manager Caring Behavior Questionnaire [23]. The demographic information included sex, age, years of work, title, marital status, educational level, hospital rank, nature of the hospital, whether the hospital has carried out humanistic care, department, training on humanistic care, the level of humanistic care training, familiarity with the humanistic care, the emphasis on humanistic care, relationship with colleagues, family support for work, the passion for nursing, and job satisfaction. Wang, Lu, and Li developed the Nurse Manager Caring Behavior Questionnaire. There are 36 items reflecting three dimensions: (a) humanistic care management and concept, (b) humanistic care environment and policy, and (c) humanistic care quality and training (the 5-level Likert scale, out of a possible 180 points). A higher score reflects better care behaviors. After feeding the data into the computer using an SPSS 27.0 program, the overall Cronbach's alpha coefficient of the questionnaire was 0.985, indicating that the questionnaire had good internal consistency. The original author has permitted the questionnaire to be used in this study.

The interview guidelines were developed based on Swanson's caring theory. Before the formal interview, a pre-trial survey was conducted and audiotaped. The resulting interviews will be transcribed verbatim and used to refine the interview script. Table 2 lists the outline of the interview for the final version.

**Table 2:** Interview questions and the corresponding main topics

Main topics	Interview questions
Introductory questions	• How do you understand humanistic care? • How do you feel about the nurse managers' caring behaviors for clinical nurses? • Does the hospital have any training or activities on how nurse managers care for clinical nurses?
Knowing	• Do you know the basic information of your subordinates? • In daily work, can you detect the emotional changes of your subordinates and take further action?
Being with	• What measures do you take to make your subordinates feel cared for and accompanied by you? • Do you engage in private conversations with your subordinates and encourage them to express their true feelings?
Doing for	• Have you offered your subordinate any help? What difficulties did you encounter in this process?
Enabling	• Do you give your subordinates timely feedback and suggestions on their concern? • For clinical nurses, what measures would you take to improve their perceptions capabilities of caring?
Maintaining belief	• How do you help clinical nurses maintain a positive attitude in their daily work? • How do you encourage your subordinates to stick with something or strive to achieve a goal?

### Data collection

During Phase I (July to August 2022), questionnaires are distributed and collected via the Electronic "Questionnaire Star" network platform (https://www.wjx.cn/). Before conducting the survey, consent and cooperation were obtained from the hospital. The purpose of the survey and the questionnaire filling method were explained in detail to the research objects. After getting the consent, the formal questionnaire was filled out. The uniqueness and authenticity of the questionnaire filling were ensured by setting a single IP address authority. In this stage, the current situation of the caring behavior of nurse managers towards clinical nurses was evaluated.

In Phase II (from August to September 2022), semi-structured interviews were conducted to explore the experiences of nurse managers regarding humanistic care for clinical nurses. The study encompassed 23 provinces and 4 municipalities in China. One hospital was selected randomly from each of the 27 localities included in the Phase I quantitative study, along with one nurse manager from each hospital. The researcher first determined the specific time of the interview with the participants, face-to-face interviews with participants in the same city as the researcher, and telephone interviews outside the city. The duration of the in-depth interviews lasted 30–40 min. Before interviewing participants, researchers had to obtain consent from the participants, explain the purpose of the study, and emphasize that they were free to withdraw at any point. Data saturation was reached during the interview with the 22 nurse managers. Nevertheless, we interviewed nurse managers in the remaining five of the 27 localities to ensure that we identified all the underlying themes [24].

### Statistical analysis

This study will integrate and report qualitative and quantitative data using a narrative approach [25]. This required analyzing and reporting the results from each phase separately, followed by combining complementary findings. Specifically, we combined the quantitative component’s measure of caring behavior among nurse managers with qualitative themes and sub-themes demonstrating perceptions and experiences regarding humanistic care management. It was necessary to merge these results because relying solely on quantitative measures does not convey the nuanced perspectives on implementing caring management.

In Phase I, the quantitative data were analyzed using IBM SPSS Statistics 27.0. Descriptive statistics were used to describe continuous variables, and percentages were used to describe categorical variables (participants’ demographic characteristics). Mean and standard deviation were considered to describe caring behavior scores. The scoring rate was computed as (actual score/total score) *100%. Furthermore, mean comparisons for the continuous variables were performed using independent t-tests (for comparisons between two groups). The comparison of multiple sample means was performed by one-way analysis of variance (ANOVA) if the values conformed to the normal distribution and homogeneity of variance; otherwise, the rank sum test was used. The test level α = 0.05 was used, and *P* < 0.05 was considered statistically significant.

In Phase II, the qualitative data were analyzed using NVivo 12. The audio was promptly transcribed verbatim, edited by the researcher, and supplemented with notes taken during the interview. The information was coded and entered into NVivo 12, and the text version was sent to the respondents to confirm the accuracy of the data. The researchers used thematic analysis [26]. To assure transparency and reliability, two researchers (LLL and YXF) independently coded all transcripts systematically. If the opinions were not unified, they were resolved through discussion with members of the research team.

### Ethical approvals

This research was approved by the Ethical Committee of Tongji Medical College, Huazhong University of Science and Technology (project No.2022-S161). Participants received an electronic notification before starting the survey, stating that completing the survey implies their agreement to provide informed consent. Participants were voluntary, anonymous, and could be withdrawn at any time. Furthermore, any publication will not contain any information that could be used to identify individual participants.

## Results

### Phase 1: quantitative survey

#### Current status of nurse managers' caring behaviors for clinical nurses

A questionnaire survey was used to investigate the caring behaviors of 2022 nurse managers from 101 hospitals in 23 provinces and 4 municipalities in China. The mean (± SD) age of the participants was 41.47 ± 6.061 years, with 98.3% of the participants female. Table 3 lists their demographic information. We observed that the overall level of caring behaviors exhibited by nurse managers was rated moderately good, with a total scoring rate of 88.55%. The overall score was 161.19 ± 20.68; the first dimension (humanistic care management and concept) received 53.39 ± 7.06, the second dimension (humanistic care environment and policy) received 71.78 ± 9.55, the third dimension (humanistic care quality and training) had 36.01 ± 4.93. Table 4 lists the detailed information.

**Table 3:** The demographic information of quantitative survey (*N* = 2022)

Variable	Classification	*N*	%
Sex	Male	35	1.7
	female	1987	98.3
Age	Mean (SD)	41.47(6.061)	-
	Range	24–59	-
Years of work	Mean (SD)	20.65(7.029)	-
	Range	2–41	-
Years of working as a nurse manager	Mean (SD)	8.40(5.302)	-
	Range	1–35	-
Title	Nurse practitioner	92	4.5
	Nurse-in-charge	1081	53.5
	Deputy chief physician	774	38.3
	Chief physician	75	3.7
Marital status	Unmarried	65	3.2
	Married/Remarried	1898	93.9
	Divorced	51	2.5
	Death of a spouse	8	0.4
Educational level	Junior college	99	4.9
	Undergraduates	1753	86.7
	Master	169	8.4
	Doctor	1	0.0
Hospital Rank	Secondary hospital	200	9.9
	Tertiary hospital	1822	90.1
Nature of the hospital	The specialized hospital	260	12.9
	General hospital	1762	87.1
Whether the hospital has carried out humanistic care	No	938	46.4
	Yes	1084	53.6
Department	Pediatric	122	6.0
	The department of obstetrics and gynecology	173	8.6
	The emergency department	56	2.8
	Department of geriatrics	57	2.8
	Outpatient service	53	2.6
	Internal medicine	541	26.8
	Surgical	54	2.7
	The operating room	496	24.5
	ophthalmology and otorhinolaryngology	57	2.8
	oncology	92	4.5
	Intensive Care Unit (ICU)	110	5.4
	other	211	10.4
Training on humanistic care	No	404	20.0
	Yes	1618	80.0
The level of humanistic care training	No	404	20.0
	Hospital grade	888	43.9
	School grade	27	1.3
	Province grade	394	19.5
	Country grade	282	13.9
	Other	27	1.3
Familiarity with the humanistic care	Very unfamiliar	21	1.0
	Unfamiliar	137	6.8
	General	718	35.5
	Familiar	790	39.1
	Very familiar	356	17.6
The emphasis on humanistic care	Think very little of	20	1.0
	Think little of	90	4.5
	General	486	24.0
	Attach importance to	620	30.7
	Attach very importance to	806	39.9
Relationship with colleagues	Very unharmonious	2	0.1
	Not harmonious	34	1.7
	General	110	5.4
	harmony	537	26.6
	Very harmonious	1339	66.2
Family support for work	Very unsupportive	5	0.2
	Unsupportive	30	1.5
	General	107	5.3
	supportive	433	21.4
	Very supportive	1447	71.6
The passion for nursing	Very dislike	2	0.1
	Dislike	40	2.0
	General	138	6.8
	like	610	30.2
	Very like	1232	60.9
Job satisfaction	Very dissatisfied	5	0.2
	Dissatisfied	54	2.7
	General	220	10.9
	Satisfied	779	38.5
	Very satisfied	964	47.7

**Table 4:** Total scale and dimension scores of nurse managers' care behaviors for nurses (*N* = 2022)

Dimension	Range	$\overline{X }$±s	Scoring rate (%)
1.Humanistic care management and concept	12–60	53.39 ± 7.06	88.99
1.1 Guarantee of system	8–40	35.28 ± 4.82	88.20
1.2 Humanistic ideas	4–20	18.11 ± 2.52	90.57
2.Humanistic care environment and policy	16–80	71.78 ± 9.55	89.73
2.1 Working environment	3–15	13.66 ± 1.81	91.06
2.2 Emotional support	7–35	31.69 ± 4.20	90.55
2.3 Care policy	6–30	26.43 ± 4.00	88.11
3.Humanistic care quality and training	8–40	36.01 ± 4.93	90.03
3.1 Quality cultivation	4–20	17.91 ± 2.56	89.56
3.2 Education and training	4–20	18.10 ± 2.51	90.50
Total dimension	36–180	161.19 ± 20.68	89.55

#### The relationship between the general information and the total score of nurse managers' caring behaviors

Different sexes, different hospital ranks, and whether or not to participate in humanistic care training were found to be significantly associated with caring behavior scores (F = 4.348, F = -3.418, F = -8.292, *P* < 0.05) (Table 5). There were also differences in the caring behavior scores of nurse managers in different departments (F = 2.455, *P* < 0.01), among which the three departments with the highest scores were oncology, obstetrics and gynecology, and internal medicine. Furthermore, the relationship with colleagues, satisfaction with work, familiarity with the humanistic care, family support for work, and the emphasis on humanistic care are also significant to the score of caring behaviors (*P* < 0.05) (Table 5).

**Table 5:** Relationship among the nurse manager’s caring behaviors score and Socio-demographic Characteristics (*N* = 2022)

Characteristics	Classification	the nurse manager’s caring behavior score		
		$\overline{X }$±s	T/F/H	*P*
Sex	Male	153.97 ± 20.94	4.348	0.037^*^
	female	161.32 ± 20.66		
Title	Nurse practitioner	151.38 ± 31.84	5.767	0.124
	Nurse-in-charge	160.69 ± 21.16		
	Deputy chief physician	163.19 ± 17.99		
	Chief physician	159.83 ± 18.71		
Marital status	Unmarried	160.03 ± 19.69	2.213	0.085
	Married/Remarried	161.35 ± 20.72		
	Divorced	159.63 ± 19.39		
	Death of a spouse	143.25 ± 22.37		
Educational level	Junior college	156.49 ± 24.66	4.657	0.199
	Undergraduates	161.12 ± 20.72		
	Master	164.56 ± 16.93		
	Doctor	180.00 ± 0.00		
Hospital rank	Secondary Hospital	156.26 ± 22.52	-3.418	0.001^**^
	Tertiary Hospitals	161.73 ± 20.40		
Nature of the hospital	The specialized hospital	160.45 ± 20.14	0.383	0.536
	General hospital	161.30 ± 20.76		
Whether the hospital has carried out humanistic care	No	157.03 ± 21.84	-9.038	< 0.001^**^
	Yes	164.79 ± 18.90		
Department	Pediatric	159.90 ± 21.74	2.455	0.005^**^
	The department of obstetrics and gynecology	163.19 ± 19.10		
	The emergency department	157.25 ± 20.63		
	Department of geriatrics	162.00 ± 16.82		
	Outpatient service	157.42 ± 19.92		
	Internal medicine	162.73 ± 20.55		
	Surgical	158.91 ± 18.66		
	The operating room	161.94 ± 21.19		
	ophthalmology and otorhinolaryngology	162.63 ± 20.24		
	oncology	163.46 ± 19.28		
	Intensive Care Unit (ICU)	153.95 ± 23.44		
	other	159.36 ± 20.44		
Training on humanistic care	No	152.46 ± 24.69	-8.292	< 0.001^**^
	Yes	163.37 ± 18.94		
The level of humanistic care training	No	152.64 ± 24.69	69.616	< 0.001^**^
	Hospital grade	163.06 ± 18.78		
	School grade	162.56 ± 20.61		
	Province grade	163.86 ± 17.45		
	Country grade	163.86 ± 21.31		
	Other	162.30 ± 18.35		
Familiarity with the humanistic care	Very unfamiliar	140.10 ± 35.96	372.730	< 0.001^**^
	Unfamiliar	155.12 ± 21.79		
	General	152.79 ± 21.56		
	Familiar	164.72 ± 17.58		
	Very familiar	173.90 ± 13.46		
The emphasis on humanistic care	Think very little of	141.30 ± 37.55	360.565	< 0.001^**^
	Think little of	152.73 ± 24.88		
	General	150.78 ± 21.82		
	Attach importance to	159.79 ± 18.09		
	Attach very importance to	169.99 ± 16.46		
Relationship with colleagues	Very unharmonious	108.00 ± 101.82	376.841	< 0.001^**^
	Not harmonious	142.03 ± 26.67		
	General	140.62 ± 25.99		
	harmony	151.97 ± 18.83		
	Very harmonious	167.15 ± 17.48		
Family support for work	Very unsupportive	135.40 ± 63.27	310.065	< 0.001^**^
	Unsupportive	134.37 ± 31.89		
	General	141.16 ± 22.81		
	supportive	151.79 ± 20.36		
	Very supportive	166.13 ± 17.50		
The passion for nursing	Dislike it very much	89.00 ± 74.95	365.287	< 0.001^**^
	Dislike	143.50 ± 31.98		
	General	141.53 ± 22.21		
	like	154.24 ± 19.19		
	Very like	167.53 ± 17.38		
Job satisfaction	Very dissatisfied	107.80 ± 43.90	497.994	< 0.001^**^
	Dissatisfied	145.19 ± 26.60		
	General	142.58 ± 24.19		
	Satisfied	156.98 ± 17.54		
	Very satisfied	170.02 ± 16.37		

^*^p < 0.05

^**^p < 0.01

### Phase 2: qualitative survey

#### Basic information about interviewees

A total of 27 nurse managers (5 females and 22 males) agreed to be interviewed. Their age ranged from 36 to 50, with a working time of 12 to 30 years in hospitals. Their academic qualifications were 20 bachelor's degrees and seven postgraduate degrees. They came from different departments, including the intensive care unit, pediatric internal medicine department, pediatric intensive care unit, obstetrics and gynecology, and others. To maintain the anonymity of each participant, they were labeled with a unique code. The 27 nurse managers were identified as A1-27.

#### Nurse Managers’ experiences of caring behaviors towards clinical nurses

Transcripts of nurse managers’ views and experiences of caring behaviors toward clinical nurses produced eight categories. Eventually, three themes were included, which implied the capacity of nurse managers and clinical nurses, the opportunity to implement humanistic care, and motivation to implement humanistic care (Table 6).

**Table 6:** Illustration of developing categories and identifying themes

Themes	Categories
Theme 1: Capacity of nurse managers and clinical nurses	Capacity of nurse managers
	Capacity of clinical nurses
Theme 2: Opportunity to implement humanistic care	A cohesive working environment Clear role definition Provide professional training opportunities
Theme 3: Motivation to implement humanistic care	Nurse managers perform an exemplary role Establish a clear reward and punishment mechanism Enhance the sense of collective honor and self-mission for clinical nurses

### Theme 1: capacity of nurse managers and clinical nurses

One of the most important themes identified was the “capacity of nurse managers and clinical nurses” derived from categories. The participants argued that effective caring behaviors demand continuous updating of professional knowledge and skills for nurse managers, including knowledge of the humanities, the ability of empathic communication and timely feedback, and nursing management ability. On the other hand, the interviewees showed that it was also crucial for clinical nurses to strengthen their caring perception ability and knowledge of nursing management concepts.

### Category 1: capacity of nurse managers

Most participants considered the capacity of caring as an important factor. Mastering the knowledge of humanistic care was the foundation for implementing humanistic care, which was helpful to help them understand the value of human life and form professional caring values.

*We will often carry out relevant training…… It (humanistic care training) can be in the form of a narrative or PPT. Only if you have the relevant knowledge can you understand what is going on and what you need to do (A11).*

Empathy is a very important factor in promoting a good relationship between nurse managers and clinical nurses. Participants expressed empathic communication skills, and timely feedback during communication was also highly concerned.

*Listen first, take the initiative to communicate with him, and then give him some helpful advice according to the actual situation or act as a bridge between them (colleagues)…… It would help if you put yourself in her shoes so that you would not be repelled when communicating (A10).*

### Category 2: capacity of clinical nurses

For nurse managers and clinical nurses, caring is a mutual behavior. Most nurse managers stressed that enhancing the caring perception ability of clinical nurses played an important role in accepting nurse managers' caring.

*Our department will organize a dinner party every month, during which we will play games, which will make them happy to communicate. The more care clinical nurses feel, the stronger their ability to care for others (A8).*

### Theme 2: opportunity to implement humanistic care

The opportunity to implement humanistic care is another main theme extracted in the present study, derived from categories of a cohesive working environment, clear role definition, and professional training opportunities.

### Category 1: a cohesive working environment

**Fig. 3:**
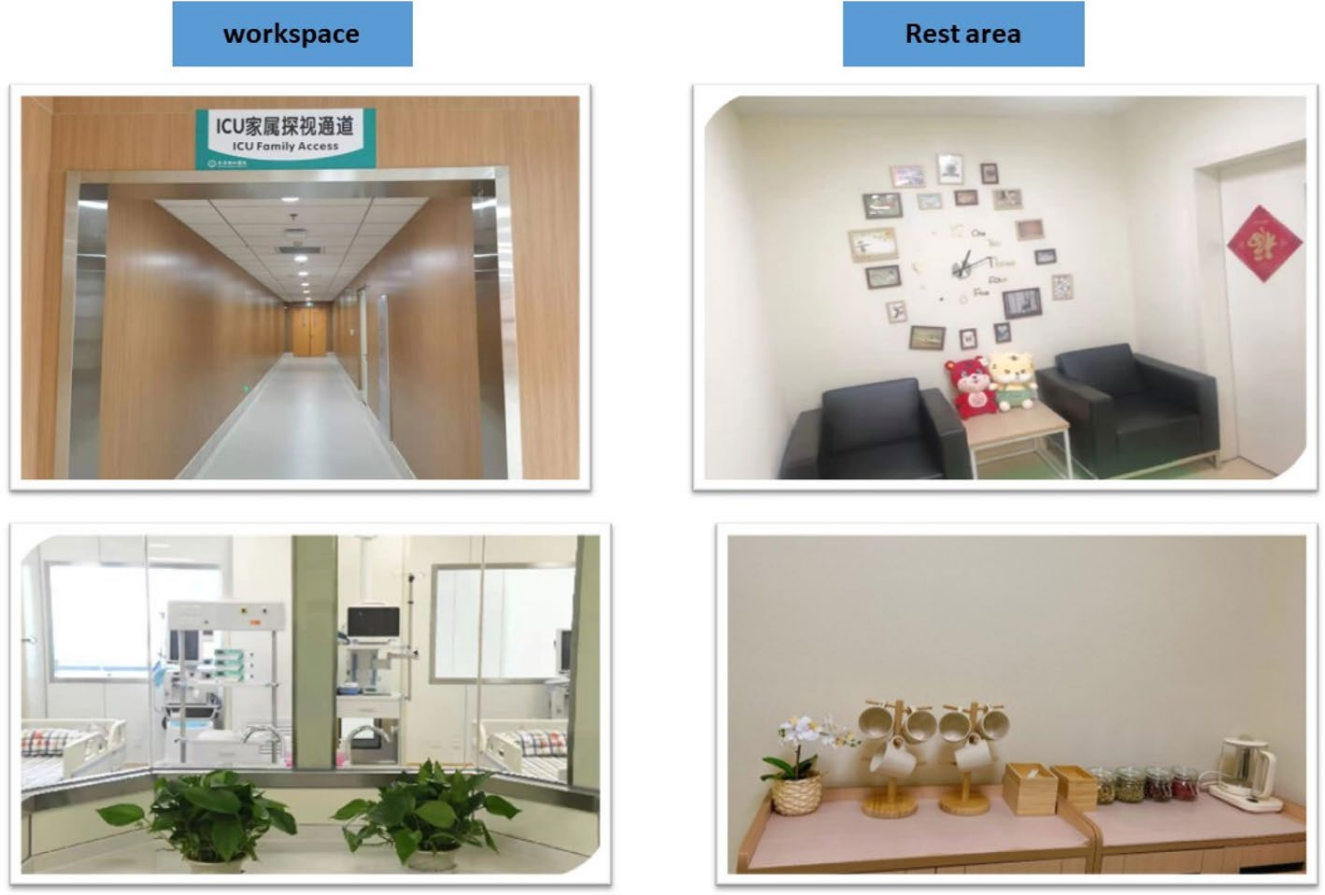
The environment of the workspace and rest area inside the department. Photograph: “In contrast to the white walls of the working area, the walls of the sitting area have been replaced with pale yellow wallpaper!” Examples of the wallpapers result in the nurse manager's caring for the clinical nurse

Almost all participants stressed the necessity of establishing a cohesive working environment. A better organizational atmosphere in the workplace is more conducive to the implementation of caring management. Figure 3 depicts that participants also shared the environment of the work and rest areas in the department.

*The working atmosphere of our department (ICU) is particularly good, and nurses are under great pressure due to the particularity of patients in our department. I will separate the decorative areas of the work area and the rest area, so that they can relax in time, which is also a kind of care (A5).*

### Category 2: clear role definition

The nurse managers showed that they should clarify their role orientation when implementing caring behaviors at work. They must act as leaders, collaborators, and coordinators and switch between them.

*As a leader, just because you work longer with someone, you cannot always be close to him or hand in hand with him in the workplace. This behavior should try not to appear in this department; you should let others think you are the center of the circle and then the point of their circle, implying that the distance between the nurse manager and everyone should be the same. This will allow for better care management, because the clinical nurses will be more accepting and feel that you are treating them fairly (A5).*

### Category 3: provide professional training opportunities

Most participants argued that professional humanistic care training was necessary for clinical nurses and nurse managers.

*I have participated in the training of the hospital, but the knowledge and ideas are constantly updated; I think regular training is necessary……(A11).*

*In addition to humanistic care training for us (nurse managers), clinical nurses also need to participate because only they understand it to accept better, which itself is a mutual process……(A16).*

### Theme 3: motivation to implement humanistic care

Motivation to implement humanistic care is the final theme extracted in the study, derived from categories of nurse managers performing an exemplary role, establishing a clear reward and punishment mechanism, and enhancing the sense of collective honor and self-mission of clinical nurses.

### Category 1: nurse Managers perform an exemplary role

Most nurse managers stated that performing an exemplary role is helpful for clinical nurses to establish their values and pass on care.

*For being short-handed, I came out to help, and they felt I worked with them to make them feel that we were a group and not just someone standing around dictating to them. When you rush to do these things, the nurses can actually feel that you care about them (A5).*

### Category 2: establish a clear reward and punishment mechanism

Most participants argued that establishing a clear reward and punishment mechanism was essential to implementing effective caring behaviors because their enthusiasm would increase through the reward and punishment mechanism.

*Our hospital also has a regular survey on the nurse managers to understand the evaluation of clinical nurses on the nurse managers, which is also an important basis for the nursing department leaders to score the nurse managers (A1).*

### Category 3: enhance the sense of collective honor and self-mission

Most participants emphasized that clinical nurses need to enhance the sense of collective honor and self-mission to form an effective spiritual motivation. Only when nurse managers and nurses get along well can the work be better promoted and the development of the department is promoted.

*Enhance collective sense of honor and self-mission to better implement care management. They know we are whole and that my decisions help them grow (A7).*

### Merged results

We merged data from the caring behavior questionnaire with qualitative sub-themes demonstrating the influencing factors of implementing caring management. Significant findings from the caring behavior questionnaire related to the subthemes of a cohesive work environment and professional training opportunities. The questionnaire found that the caring behavior scores of nurse managers with humanistic care training were higher than those of nurse managers without training, and the same goes for harmonious relationships with colleagues and caring behavior scores. These complement respondents’ reports that they believed humanistic care training was important and harmonious work climates more conducive to implementing caring management.

## Discussions

**Fig. 4:**
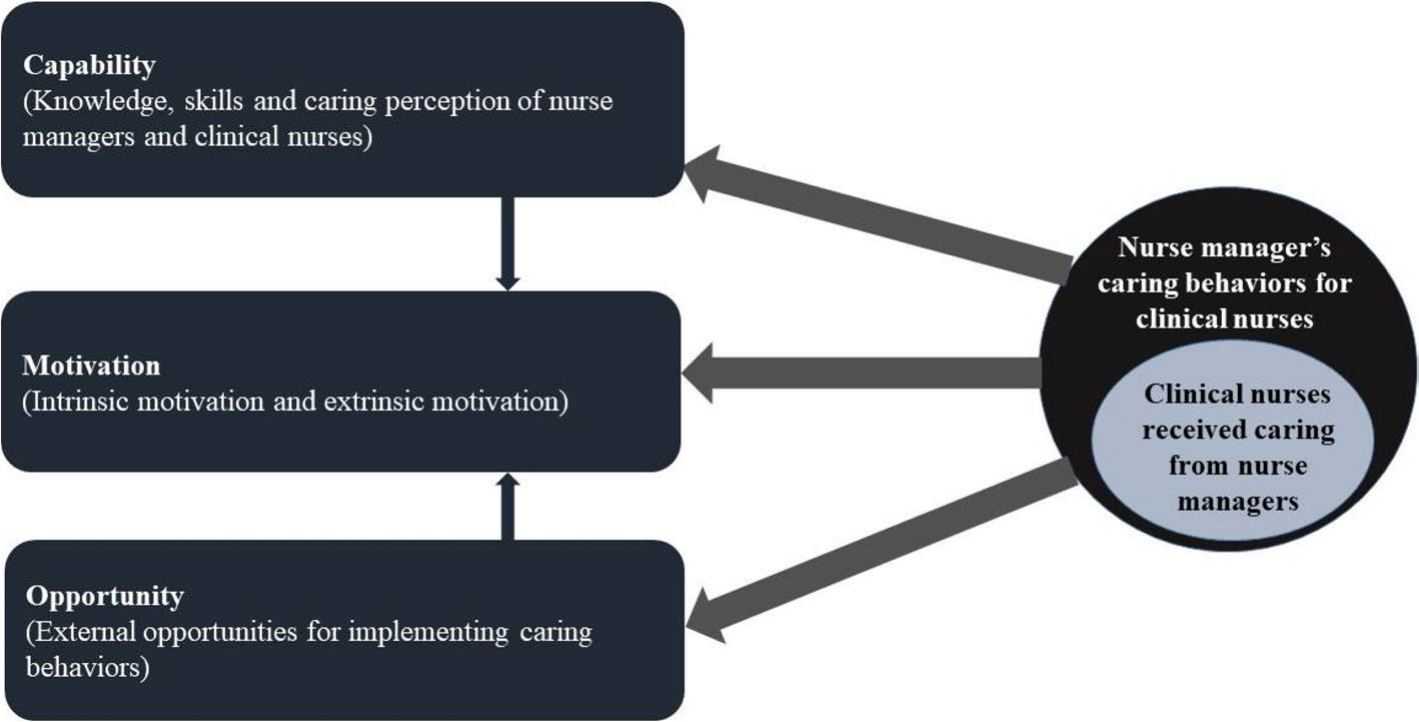
Caring behaviors framework among nurse managers towards clinical nurses

This study was designed to investigate the level of nurse managers' caring behaviors and barriers and facilitators related to implementing humanistic care. The overall scoring rate of Chinese nurse managers' caring behaviors was 89.55%, which indicated good caring behavior. Informed by the COM-B framework, we found that the capability of nurse managers and clinical nurses to provide/receive humanistic care, opportunity, and motivation to implement humanistic care tip the scales whether they effectively implement humanistic care management. Please refer to Fig. 4. We also found that caring is an interactive behavior and that building trust is important for providing caring and enabling clinical nurses to accept the care. The ‘Caring behaviors framework among nurse managers towards clinical nurses’ (Fig. 4) developed in the study would provide a basis for the comprehensive intervention.

Findings from the caring behavior questionnaire were congruent with the qualitative data that emphasized the opportunities to implement humanistic care, including a cohesive working environment and professional training. As indicated by the questionnaire, nurse managers who are more familiar with the humanistic care domain, have experienced professional training, and have a more harmonious relationship with colleagues have a higher caring behavior score. These preliminary quantitative results were supported by emergent themes from the interviews, which showed that nurse managers' capacity and opportunities to implement humanistic care were important factors affecting their caring behavior. The motivation of the nurse managers to implement humanistic care from the interview was also an important influencing factor. Still, this point cannot be reflected in the analysis of the quantitative questionnaire. Qualitative results revealed the influencing factors of humanistic care based on the COM-B model [18]. The findings help bridge the gaps in research and practice and further promote and develop humanistic care management in hospitals.

This study showed that over half, 56.7%, of participants was familiar with humanistic care. They acquired knowledge and skills about humanistic care. Whether caring management can be implemented successfully, the capability of nurse managers is vital. Regarding nurse managers, enhancing knowledge of the humanities could give the basic theoretical guidance to implement caring behaviors [27]. Empathy refers to whether a person can put themself in the other person's position, which is an important condition for establishing a good interpersonal relationship [28]. Empathy refers to whether a person can put himself or herself in the other person's position, which is the most important condition for establishing a good interpersonal relationship [29]. If nurse managers can correctly use empathic communication to transform subordinates from passive obedience to active cooperation, they can fully mobilize their work and create a cohesive unit [30].

In this study, the scores of caring behaviors were associated with different levels of relationships with colleagues, job satisfaction, and whether they had experienced humanistic care training. The qualitative research also proves the important role of opportunities in humanistic care, including a harmonious environment, clear role definition, and professional training. Researchers showed that creating a harmonious and caring atmosphere is essential for humanistic care [31]. On another scale, nurse managers clear their role definitions, which are the overarching premise to perceive and upgrade their fulfillment of responsibilities [32]. As the nursing leader, the controller of nursing quality, and the coordinator of department work, the nurse managers must be able to switch roles at any time according to different scenarios. Nurse managers should strengthen clinical nurses' humanistic quality and professional education in their roles as nurse managers. Besides, these findings prove that nurse managers were proactive in implementing humanistic care behaviors. They can implement better humanistic care only when they fully know the importance of caring management.

However, this study also had some limitations. First, a random sample was not used in this study, although it involved 101 hospitals in 23 provinces and 4 municipalities in China, representing the national situation. Second, due to COVID-19, some participants collected data through telephone interviews, which may not be as reliable as face-to-face interviews. We also recorded the entire process, transcribed it into text, and gave it to the interviewees for verification to ensure the reliability of the results as much as possible. Finally, we did not get additional qualitative information from the clinical nurses. Future researchers should consider this to have more detailed descriptions.

### Implication of research

This study emphasizes the importance of the capability, opportunity, and motivation to implement humanistic management and facilitate the translation of research findings into clinical practice. Healthcare organizations could provide targeted education and training for nurse managers and clinical nurses based on their competencies and needs, improving the care management abilities of nurse managers and clinical nurses' perceived care abilities. Nurse managers must proactively engage with clinical nurses, suggesting that empathic communication and timely feedback are crucial to humanistic management. Organizational policies about humanistic management ought to be more available, with the working environment included in the department's quarterly assessment. Hospitals should also establish appropriate reward and punishment mechanisms. Nursing department leaders should communicate promptly and give corresponding praise or punishment to nurse managers with different scores for caring behaviors. Developing and evaluating such interventions, developing feasible intervention plans, and implementing them to verify their effectiveness are important avenues for future research.

## Conclusions

This study presented scientific data for nurse managers' perceptions and experiences of caring behaviors for clinical nurses. The results indicated that the overall level of caring behaviors of the nurse managers was moderately good. Through the experience of nurse managers, enablers and barriers to the implementation of humanistic care were derived. The findings suggest that intrinsic motivation, organizational support, and the capabilities of clinical nurses and nurse managers are critical to implementing care behaviors. Implementing successful humanistic care requires common efforts at the individual and organizational levels.

### Supplementary Information

**Additional file 1.** Good Reporting of a Mixed Methods Study (GRAMMS) Checklist.

## Data Availability

The datasets used or analyzed during the current study are available from the Corresponding author on reasonable request.

## Abbreviations

COVID-19: Corona virus disease 2019
COM-B model: Capacity, opportunity, motivation-behavior model
GRAMMS: Good reporting of a mixed methods study
